# A Differential Resonant Voltage Sensor Consisting of Piezo Bimorph and Quartz Crystal Double-Ended Tuning Fork Resonators

**DOI:** 10.3390/s19225031

**Published:** 2019-11-18

**Authors:** Zijun Huang, Leixiang Bian

**Affiliations:** School of Mechanical and Engineering, Nanjing University of Science and Technology, Nanjing 210094, China

**Keywords:** voltage sensor, double-ended tuning fork (DETF), piezo bimorph, resonant sensor

## Abstract

A differential resonant voltage sensor with frequency output was developed by bonding two quartz crystal double-ended tuning forks (DETFs) on both sides of a piezo bimorph. The applied voltage induced tensile and compression deformation in the upper and bottom layers of the piezo bimorph, which caused the resonant frequency of the dual DETFs to increase and decrease, respectively. In this case, the differential output of the resonance frequencies of the dual DETFs greatly reduced the effect of temperature drift. In addition, the input resistance of the piezo bimorph reached a few hundred GΩ, which caused almost no influence on the DC voltage under test. The fabricated device showed a linear characteristic over its measurement range of ±700 V with a sensitivity of 0.75 Hz/V, a resolution of 0.007% (0.1 V) and hysteresis of 0.76% of the full range. The quality factor of the DETFs was about 3661 (in air). This novel resonant voltage sensor with its extremely low power consumption is promising for measuring or monitoring DC voltage in various fields.

## 1. Introduction

Voltage/electric field sensors are widely used in a variety of applications such as power grids [1], Internet of Things (IoT) [2], oil well health monitoring [3], and even smart houses [4]. Traditional voltage measuring devices include resistance voltage dividers, capacitive voltage dividers, and electromagnetic voltage transformers. Capacitive dividers and electromagnetic voltage transformers are usually used for AC voltage measurement. Resistance voltage dividers can be used for either DC or AC voltage measurement [5]. However, the self-heating effect of resistances causes additional power consumption and inaccuracy problems. 

The past few decades has witnessed the development of many novel DC/AC voltage sensors such as the optical voltage sensors (OVSs) based on a linear electro-optic effect (Pockels effect) [6], and Kerr effect [7]. In addition, the voltage sensors using piezoelectric material (Pb(Zr_1−x_Ti_x_)O_3_, PZT) as a voltage transducer and a fiber Bragg grating as a strain sensor have also been developed [8]. The voltage sensors employ electro-optical crystal or piezoelectric material and feature high input resistance of a few hundred GΩ, a small size, low weight, and immunity to electromagnetic interference. However, these OVSs need complicated photoelectric demodulation systems where measurement accuracy is easily influenced by light intensity and temperature. Xue et al. [9] reported a piezoelectric–piezoresistive coupling MEMS sensor, which could be used to measure a DC/AC electric field or voltage with a broadened frequency bandwidth of up to 100 kHz. Sasaki et al. [10,11] reported a MEMS resonator-based voltage sensor in which the resonator was designed as a floating electrode sandwiched between a high-voltage electrode and a driving electrode. The transverse electrostatic attraction force generated by the electrical field of a high DC voltage source draws the frequency change of the resonator. However, the sensor shows strong nonlinearity over its measurement range of 400 V due to the nonlinear transverse electrostatic attraction force.

This paper reports on a differential resonant voltage sensor using a piezo bimorph as a voltage transducer and two differential quartz crystal double-ended tuning forks (DETFs) as force sensors. When a voltage is applied to the piezo bimorph, the tensile and compression deformations arise in the piezo bimorph due to the inverse piezoelectric effect and are transferred to the longitudinal of the dual DETFs whose resonant frequencies are in turn caused to increase and decrease, respectively. In the configuration, the differential output of the resonant frequencies of the dual DETFs doubled the sensor’s sensitivity and greatly reduced the influence of temperature drift. The frequency shift of the sensor can be measured by a frequency counter.

## 2. The Principle and Configuration of the Differential Resonant Voltage Sensor

### 2.1. Design and Configuration of the Device

**Figure 1:**
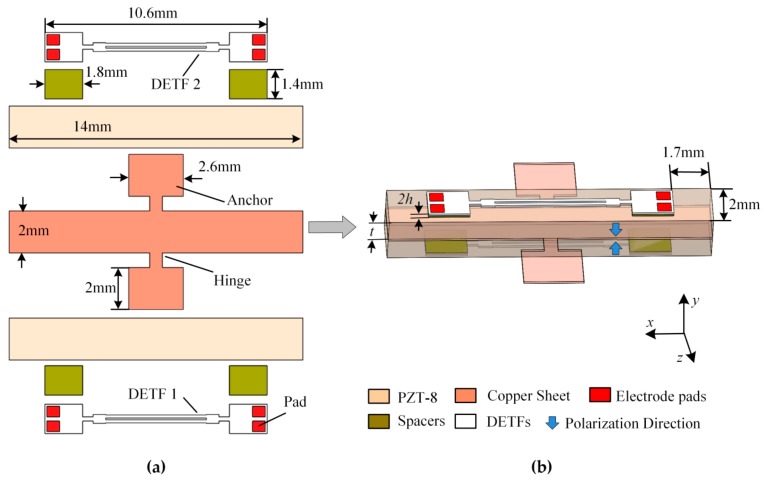
Schematic diagram of the differential resonant voltage sensor. (**a**) The components of the sensor; (**b**) the assembled sensor.

The schematic diagram of the differential voltage sensor is shown in Figure 1, including a piezo bimorph, dual DETFs and four quartz crystal spacers. The series connection mode [12] was used in the piezo bimorph fabrication, namely, two piezoelectric Pb(Zr_1−x_Ti_x_)O_3_ (PZT-8) plates with an opposite polarization direction bonded on both sides of a copper sheet by epoxy resin and an electrical voltage, which was applied across the total thickness. Two support hinges with a rectangular anchored structure was designed in the middle of the copper sheet. The two anchors were used to fix the sensor. The sizes of PZT-8 plates were 14 mm×2 mm×1 mm. The main body of the copper sheet was the size of 14 mm×2 mm×0.1 mm. The sizes of the hinges and rectangular anchors were 0.7 mm×0.6 mm and 2.6 mm×2 mm, respectively. The dual DETFs were symmetrically bonded on the upper and bottom surfaces of the piezo bimorph, respectively. In order to transfer the action force of the piezo bimorph and keep non-contact of the tines of the DETFs with the piezo bimorph, four spacers were bonded under the double ends of each DETF. The spacers were cut from a Z-cut quartz sheet with a size of 1.8 mm×1.4 mm×0.1 mm.

**Figure 2:**
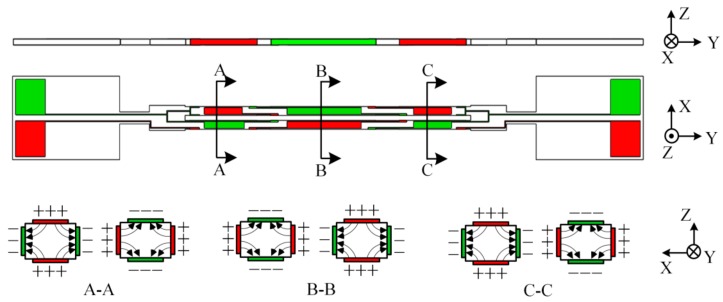
The electrode patterns of the double-ended tuning forks (DETF).

The DETF is an axisymmetric structure fabricated from a Z-cut quartz wafer. The length, width, and thickness of the DETF tines were designed along the mechanical axis (*Y*-axis), electrical axis (*X*-axis), and optical axis (*Z*-axis) of a quartz crystal, respectively. The conventional four-electrode patterns (as shown in Figure 2) were used to operate the DETF in an anti-phase flexural mode. The electrode patterns surrounding the four surfaces of the DETF tines changed direction around the location of 0.224 and 0.776 times the length of the tines where the stress values were zero [13]. More information about the design and fabrication of the DETF can be found in [13]. In this mode, the anchor zones of the DETF, which were bonded to the piezo bimorph through spacers was actually the decoupling region of the DETF. In this manner, the loss of the piezo bimorph was isolated in the piezoelectric layers, resulting in no influence on the quality factor (*Q*-factor) of the DETF. The detailed dimensions of the DETF is shown in Table 1.

**Figure 3:**
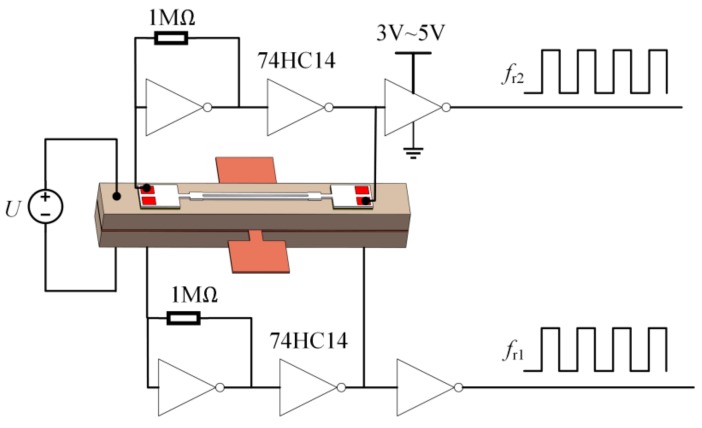
Gate oscillator circuit.

Under the action of the applied voltage, the tensile and compression stresses/strains generated in the upper and bottom layers of the piezo bimorph were transferred to the longitudinal direction of the DETFs. In turn, the resonant frequencies of the dual DETFs led to increases and decreases, respectively. The gate oscillator circuit, as shown in Figure 3, can be used to generate steady and nearly perfect square signals whose frequency is determined by the resonance frequency of the DETF [13]. The CMOS inverter with feedback resistor works as an amplifier. The DETF acts as a feedback network to determine the output frequency of the oscillator. In addition, the power consumption of the power supply to gate oscillator circuit was measured to be 150 μW (3 V, 50 μA) by a digital multimeter (Fluke 17B). The frequency shift of the square signal can be measured by a frequency counter.

With the well-known formulae of body resistance and parallel capacitance, i.e., $R=\rho_{R}d/S$ and $C=\varepsilon_{r}\varepsilon_{0}S/d\;$, where, $\rho_{R}$ and $\varepsilon_{r}$ are the resistivity and relative dielectric constant, d and S are the thickness and surface area of the PZT-8 plates, respectively, $\varepsilon_{0}$ is the permittivity of vacuum. Note that, the surface area S was equal to the electrode area of the PZT-8 plate. Taking the typical parameters from Table 2, the resistance and static capacitance of the piezo bimorph were evaluated to be >714 GΩ and about 124 pF, respectively. The high resistance and static capacitance determined the high input impedance of the piezo bimorph. This meant that the connection of the voltage sensor caused almost no influence on the DC voltage under test.

### 2.2. Theoretical Analysis and Simulation

For a clamped–clamped beam with flexural vibration, the functional relationship between the fundamental resonant frequency $f_{r}$ and the applied force F along its longitudinal direction can be expressed by [14]:

(1) $$f_{r}=\;f_{0}\sqrt{1+\gamma_{0}F\frac{l^{2}}{E_{q}I}}\;$$

where $f_{0}=\frac{\alpha_{0}^{2}}{2\pi l^{2}}\sqrt{\frac{E_{q}I}{\rho A}}$ is the fundamental resonant frequency without longitudinal force, A=hw and $I=hw^{3}/12$ are the cross-sectional area and the second moment of inertia, respectively; l, h, and w are the length, thickness, and width of the beam, respectively; $E_{q}$ and ρ are the Young’s modulus and mass density of the quartz beam, respectively; The constant $\alpha_{0}$ and $\gamma_{0}$ are 4.730 and 0.0245775, respectively.

In tensile and compression mode, the force F gets a positive and negative sign, respectively. Then the differential frequency output of the sensor can be written as:

(2) $$\Delta f=f_{r1}-f_{r2}$$

where, $f_{r1}$ and $f_{r2}$ are the resonant frequency of the dual DETFs of unit 1 and unit 2, respectively. The differential frequency output was expected to double the sensitivity of the sensor and greatly reduce the effect of the temperature drift.

The structure of the device can be analyzed using the Euler–Bernoulli model [15]. Due to the symmetry, the neutral layer and neutral axis were at the mid-thickness and the geometric center of the structure, respectively. As the neutral axis acts as a level-arm and determines the stress distribution within the structure $\int_{0}^{a_{3}}ybE_{a}(yk\;+\Lambda )$ , the bending moments acting on the piezo bimorph and the DETFs can be determined by integrating the stress over the thickness [15]. The integration margins were abbreviated with $a_{i}$, where $a_{1}=t+2h$, $a_{2}=t+h$, $a_{3}=t$ and $a_{4}=-t$, t is the thickness of the individual PZT-8 plate, h is the thickness of DETF. As the thickness of the copper sheet was much smaller than that of the PZT-8 plates, the influence of the copper sheet was neglected for a simplified analysis. Therefore, the moment $M_{a}$ generated by the piezo bimorph was given by:

(3) $$M_{a}\;=\;\int_{0}^{a_{3}}ybE_{a}(yk\;+\Lambda )dy\;+\;\int_{a_{4}}^{0}ybE_{a}(yk\;-\Lambda )dy=\;\frac{2}{3}\;E_{a}bt^{3}k\;+\;E_{a}bt^{2}\Lambda$$

where, *E_a_* is the elasticity modulus of PZT-8, *k* is the curvature of the piezo bimorph, *b* is the width of the PZT-8 plates, and $\Lambda \;=\;d_{31}E\;=\;d_{31}\cdot U/2t$ is the strain of the PZT-8 plates under voltage *U*, *d*_31_ is the piezoelectric constant of the PZT-8. From (3), the integration result revealed that the moment *M_a_* was composed of two distinct parts, the first part related to a bending term arising from the curvature induced into the piezo bimorph, and the second part related to the induced strain actuation.

Because of the symmetry of the sensor structure, the total moment *M_b_* acting on the dual DETFs was twice the moment *M*_*b*1_ acting on the upper DETF. By integrating the stress over the thickness of the DETFs, the total moment *M_b_* was obtained as shown below:

(4) $$M_{b}\;=\;2M_{b1}\;=\;2\int_{a_{2}}^{a_{1}}yE_{q}2wyk\;dy\;=\;\frac{4}{3}E_{q}hw(7h^{2}\;+\;9ht\;+\;3t^{2})\kappa$$

As the moments of the piezo bimorph and the dual DETFs are balanced, one gets:

(5) $$M_{a}=M_{b}$$

Substituting (3) and (4) into (5), and solving for the curvature κ yields:

(6) $$\kappa =\frac{3E_{a}btd_{31}}{8E_{q}hw\left(7h^{2}+9ht+3t^{2}\right)-4E_{a}bt^{3}}U$$

The upper and bottom layers of the piezo bimorph always undergo the opposite deformation (tensile or compression). The force F transferred to the beams of the dual DETFs can be given by:

(7) $$F=\pm E_{q}\varepsilon A=\pm E_{q}\kappa \left(t+\frac{3}{2}h\right)hw$$

From (6) and (7), the force transferred to the DETFs was related to the dimension and piezoelectric constant $d_{31}$ of the piezo bimorph. Thus, the measurement range and sensitivity of the sensor can be optimized and designed by altering the dimension and material of the piezo bimorph. Taking the typical parameters of PZT-8 and quartz crystal from Table 1 and Table 2 into (1), (2), and (7), one gets that the total shift of the differential frequency output Δf is 1434 Hz over the range of ±700 V. 

**Figure 4:**
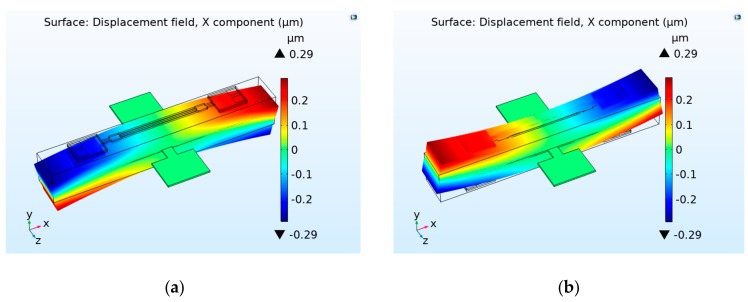
Displacement distribution of the sensor structure under the action of applied voltage of (**a**) +700 V and (**b**) −700 V.

Figure 4 shows the deformation of the sensor structure under the action of an applied voltage of ±700 V simulated by COMSOL software. As can be seen, the sensor structure generates symmetrical and anti-phase deformation on the dual-side of the neutral of the piezo bimorph under the applied voltage of 700 V and −700 V. When the voltage was 700 V, the displacement changed along the longitudinal direction of the dual DETFs, which were −0.49058 μm and 0.4907 μm, corresponding to average strains of −46.28 ppm and 46.29 ppm, respectively. Taking the simulation results into (2) and (7), the differential frequency output was calculated as 1095 Hz. This result was less than the theoretical calculation result of 1434 Hz. The difference between the two methods may have resulted from the modeling error. The theoretical calculation model ignores the existence of the copper sheet, the hinges and the anchors, which are fully considered in finite element simulation. In addition, the anisotropic parameters of the piezoelectric ceramic PZT-8 were used in the simulation, namely the piezoelectric strain constant matrix and elastic constant matrix as shown in the Appendix A. However, only the elastic modulus and strain along the longitudinal direction were considered in the theoretical calculation model.

## 3. Device Fabrication and Experiments

### 3.1. Fabrication of the Device

**Figure 5:**
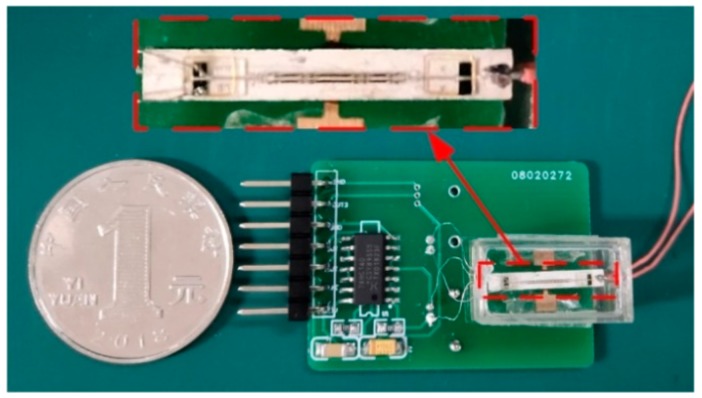
Photograph of the composite sensor with the gate oscillator circuit.

The DETFs were made from a Z-cut quartz wafer. The fabrication process was the same as that in [16]. To form a piezo bimorph, two PZT-8 plates with an opposite polarization direction were first bonded on both sides of the copper sheet. Then, the spacers and dual DETFs were symmetrically bonded on the upper and bottom surfaces of the piezo bimorph. The epoxy resin adhesive was used for the entire bonding process of the device. Figure 5 shows the photograph of the composite sensor with the gate oscillator circuit.

### 3.2. Experimental Setup

**Figure 6:**
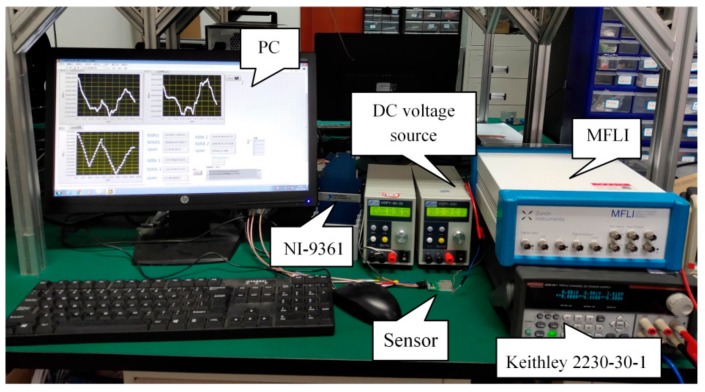
The overall view of the experimental setup.

The overall view of the experimental setup is shown in Figure 6. A precision impedance analyzer (a lock-in amplifier with impedance analyzer option, MFLI 5 MHz, Zurich Instruments, Zurich, Switzerland) was used to test the impedance characteristics of the resonant voltage sensor. A DC voltage source (1000 V, 0.2 A, Hanshengpuyuan, Beijing, China) was used to measure the response characteristics of the sensor over a high voltage range, and a precision DC power supply (Keithley 2230-30-1, Tektronix, Beaverton, OR, USA) was used to evaluate the resolution of the sensor. The frequency of the output signal from the gate oscillator was measured by a NI-9361 digital counter with LabVIEW software. An automatic system was built for the measurement of the voltage response characteristics and the resolution of the sensor by LabVIEW software. The entire experiment was carried out at room temperature and ambient air pressure.

## 4. Results and Discussions

**Figure 7:**
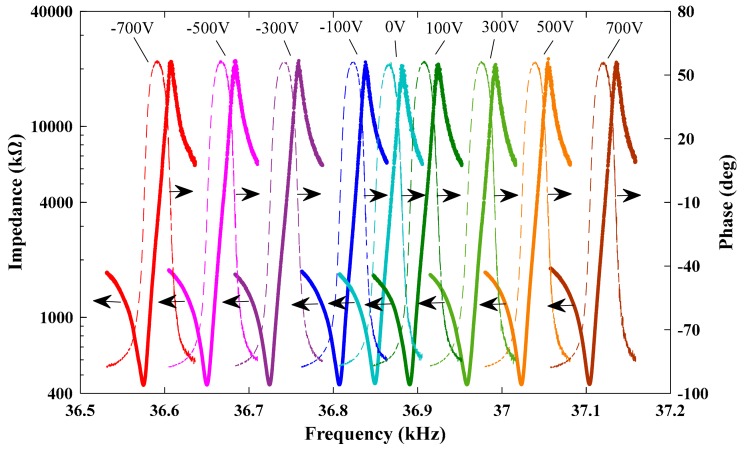
Magnitude response of the individual DETF’s impedance at various DC voltages.

**Figure 8:**
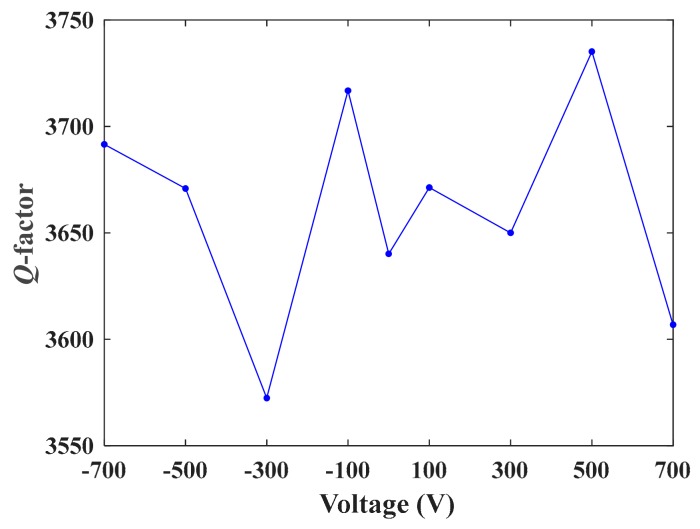
The *Q*-factor of DETF 2 as a function of DC voltage.

Figure 7 shows the impedance characteristics of the DETF 2. Obviously, the resonant frequencies clearly changed with the DC voltage. The *Q*-factor of the DETF was determined by $Q=f_{r}/\left(f_{2}-f_{1}\right)$, where, $f_{r}$ was the resonant frequency of the DETF, $f_{1}$ and $f_{2}$ were the frequencies at the half power points. The values of $f_{r}$, $f_{1}$, and $f_{2}$ were obtained from the impedance characteristics curves by the method described in [17]. Figure 8 shows the *Q*-factor of DETF 2 as a function of DC voltage. The *Q*-factor fluctuated between 3572 and 3735, with an average value of 3661. The fluctuation of the *Q*-factor may have resulted from the limited number of sweep data points of the impedance curves, which were measured by the sweep frequency method [13]. The high *Q*-factor meant that the sensor may have obtained high signal-to-noise ratio, high resolution and low power consumption.

**Figure 9:**
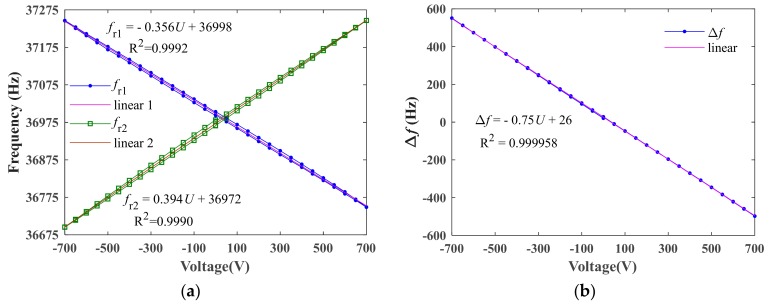
The resonant frequencies of individual DETF 1 and DETF 2 (**a**) and the differential frequency output (**b**) as a function of external DC voltage.

Figure 9a shows the resonant frequencies of DETF 1 and DETF 2 as a function of the applied DC voltage. In the range of ±700 V, the total frequency shift of DETF 1 and DETF 2 was 498 Hz and 551 Hz, respectively. The linear fitting functions were obtained as $f_{r1}=-0.356U+36998$ (linear correlation coefficient R^2^ = 0.9992) and $f_{r2}=0.394U+36972$ (linear correlation coefficient R^2^ = 0.9990). Thus, the voltage sensitivities measured by DETF 1 and DETF 2 were 0.356 Hz/V and 0.394 Hz/V, respectively. Figure 9b shows the differential frequency output of the sensor as a function of the applied DC voltage. In the differential configuration, the total frequency shift was almost doubled, being 1049 Hz. This result agreed well with the simulation result. The small difference between the experimental and simulation results may have resulted from the dimensions and parameters errors of the DETFs and the PZT-8 plates as well as the neglect of the adhesive layer in the simulation. From Figure 9b, the linear fitting function became Δf=−0.75U+26 (linear correlation coefficient R^2^ = 0.999958) showing the sensitivity of the sensor as 0.75 Hz/V. In addition, the differential output of the sensor had a non-linearity error of 3 Hz (0.29% of the full range).

**Figure 10:**
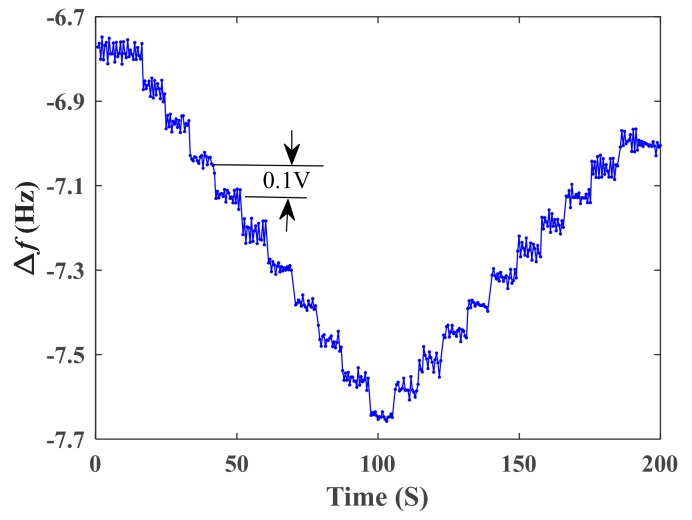
Response of the sensor to small step changes of DC voltage.

Figure 10 shows the response of the sensor to small step changes of DC voltage. The result shows that the differential frequency output of the sensor could distinguish the small step changes of DC voltage. The mean values of the differential frequency outputs ${\bar{\Delta f}}_{i}$ at the i voltage step and the step changes of the mean value ${\bar{\Delta f}}_{i}-{\bar{\Delta f}}_{i-1}$ between the adjacent steps are show in Table 3. There are 20 samples of the step changes. A two tail, one sample t-test at the 0.05 significance level (i.e., *p*-value ≤ 0.05) was used to check the resolution of the sensor. The mean value and standard deviation of ${\bar{\Delta f}}_{i}-{\bar{\Delta f}}_{i-1}$ were calculated as 0.0756 Hz and 0.01156 Hz, respectively. Then the test statistic *t* was calculated as 0.2267 . With the assumption of a *p*-value ≤ 0.05, one gets $t_{0.05}\left(19\right)=2.09$. Then, the *p*-value was calculated as 0.823 by using the t-test function in Matlab software, which was much larger than 0.05. It was shown that the resolution of the sensor could achieve 0.1 V (0.007% of the full range).

Figure 9 and Figure 10 also show the behavior of the hysteresis loop when the DC voltage increased and then decreased. From Figure 9, the maximum hysteresis of the differential frequency output was about 8 Hz (0.07% of the full range). For a voltage sensor employing piezoelectric material, the hysteresis effect is inevitable due to the time delay in domain switching [18]. It is very important to reduce the hysteresis for sensing applications employing piezoelectric materials. Usually, the lag compensation methods which have been employed in many piezoelectric actuators can be taken to reduce the hysteresis [19]. This work may be carried out in future.

**Figure 11:**
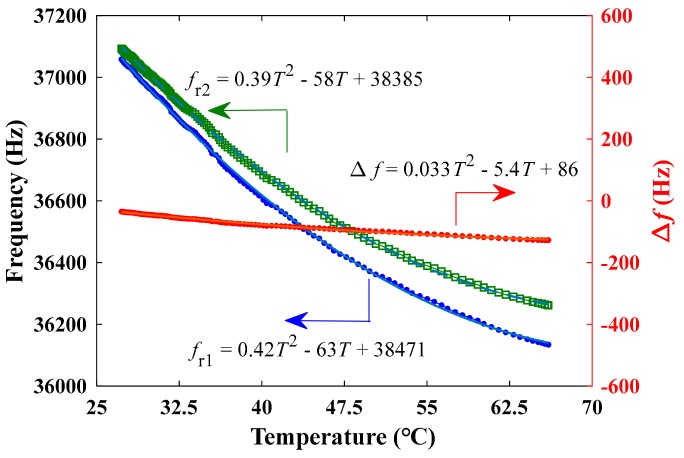
The frequencies as a function of temperature from 27 °C to 67 °C.

An initial evaluation of the temperature characteristics of the differential resonant voltage sensor was carried out. Figure 11 shows the resonant frequencies of individual DETF and the differential frequency output as a function of temperature over room temperature of 27 °C to 67 °C. As can be seen, the resonant frequency shifts measured by the DETF 1 and DETF 2 were 925 Hz and 832 Hz over the temperature range from a room temperature of 27 °C to 67 °C, while the shift in the differential frequency output reduced to 93 Hz. Furthermore, by using the polynomial fitting method, one obtains the fitting function of the curves of $f_{r1}$, $f_{r2}$ and Δf as follows:

(8) $$f_{r1}=0.42T^{2}-63T+38471$$

(9) $$f_{r2}=0.39T^{2}-58T+38385$$

(10) $$\Delta f=0.033T^{2}-5.4T+86$$

where, *T* is the temperature value. Then, the nonlinear temperature coefficients measured by the DETF 1 and DETF 2 were obtained as follows:

(11) $$df_{r1}/dT=0.84T-63$$

(12) $$df_{r2}/dT=0.78T-58$$

Where the nonlinear temperature sensitivity of the differential frequency output is as below:

(13) dΔf/dT=0.066T−5.4

From (11) to (13), the temperature coefficient in the differential mode was about an order smaller than that of the individual DETF. As the temperature changes, the induced stresses/strains in the piezoelectric and quartz materials by thermal expansion effect causes the resonant frequency of the dual DETFs to increase or decrease simultaneously. In this manner, the differential frequency output greatly reduces the influence of temperature drift. It is believed that the influence of the temperature drift can be further suppressed by the control of the fabrication process of sensors.

The main performance characteristics of the resonant voltage sensor is summarized in Table 4. The piezo bimorph/quartz DETF-based resonant voltage sensor presented here was characterized by high linear characteristic over a wide measurement range, high sensitivity, excellent resolution, high input impedance and low-power requirements. Only a few hundred microwatts of power was needed to supply the oscillator circuit.

## 5. Conclusions

A novel design and functionality of the resonant voltage sensor employing a piezo bimorph and dual DETFs was demonstrated. Under the applied DC voltage, the piezo bimorph generated tensile and compression deformation in the upper and bottom piezoelectric layers, causing the resonant frequency of the dual DETFs to increase and decrease, respectively. The differential configuration of dual DETFs doubled the voltage sensitivity of the sensor and decreased the effect of temperature drift. The fabricated device showed high sensitivity of 0.75 Hz/V and a resolution of 0.007% (0.1 V) and hysteresis of 0.76% over the linear measurement range of ±700 V. Other unique characteristics included a high Q-factor (~3661 in air), high input resistance, and low power consumption (150 μW). In future studies, we plan to develop electric field sensors based on the approach of this paper. 

## Figures and Tables

**Table 1:** The detailed dimensions of the DETF.

Description	Value
Length of tine	4.8 mm
Width of tine	0.146 mm
Thickness of tine	0.1 mm
Interval between the tines	0.1 mm
Total length of the DETF	10.6 mm
Length and width of the supporting parts of DETF	1.8 mm × 1.4 mm

**Table 2:** The typical parameters of PZT-8 and quartz crystal.

	*E* (GPa)	*ρ* (kg/m^3^)	*d*_31_ (×10^−12^ C/N)	*ε*_*r*_	*ρ_R_* (Ω m)
PZT-8	147	7600	−97	~1000	>10^10^
Quartz crystal	86	2650			

**Table 3:** Mean and variation of the differential frequency outputs for step change voltage.

Step *i*	${\bar{\Delta f}}_{i}$ (Hz)	${\bar{\Delta f}}_{i}\;-\;{\bar{\Delta f}}_{i-1}$ (Hz)	ΔU (V)	Step i	${\bar{\Delta f}}_{i}$ (Hz)	${\bar{\Delta f}}_{i}\;-\;{\bar{\Delta f}}_{i-1}$ (Hz)	ΔU (V)
0	−6.78	-	0	11	−7.58	0.065	0.9
1	−6.87	0.089	0.1	12	−7.52	0.063	0.8
2	−6.95	0.081	0.2	13	−7.44	0.068	0.7
3	−7.04	0.087	0.3	14	−7.38	0.065	0.6
4	−7.12	0.084	0.4	15	−7.32	0.066	0.5
5	−7.20	0.085	0.5	16	−7.25	0.064	0.4
6	−7.29	0.083	0.6	17	−7.19	0.063	0.3
7	−7.38	0.087	0.7	18	−7.17	0.062	0.2
8	−7.47	0.087	0.8	19	−7.06	0.068	0.1
9	−7.55	0.086	0.9	20	−7.00	0.059	0
10	−7.65	0.092	1.0				

**Table 4:** Main characteristics of the resonant voltage sensor.

Parameter	Value	As % of Full Scale
*Q*-factor (in air )	~3661	
Measurement range	±700 V	
Differential Frequency shift (for ±700 V)	1049 Hz	
Sensitivity	0.75 Hz/V	
Resolution	0.1 V	0.007
Max hysteresis	8 Hz	0.76
Max non-linearity	3 Hz	0.29
Input impedance	>714 GΩ//~124 pF	
Power consumption	150 μW (3 V, 50 μA)	

## Appendix A

The piezoelectric strain constant matrix and elastic constant matrix of the PZT-8 used in the simulation have been shown in Table A1 and Table A2, respectively.

**Table A1:** The piezoelectric strain constant matrix of the PZT-8.

0 C/N	0 C/N	0 C/N	0 C/N	$33\times 10^{-11}\;C/N$	0 C/N
0 C/N	0 C/N	0 C/N	$33\times 10^{-11}$ C/N	0 C/N	0 C/N
$-97\times 10^{-12}$ C/N	$-97\times 10^{-12}$ C/N	$22.5\times 10^{-11}$ C/N	0 C/N	0 C/N	0 C/N

**Table A2:** The elastic constant matrix of the PZT-8.

147 GPa	81 GPa	81 GPa	0 GPa	0 GPa	0 GPa
81 GPa	147 GPa	81 GPa	0 GPa	0 GPa	0 GPa
81 GPa	81 GPa	147 GPa	0 GPa	0 GPa	0 GPa
0 GPa	0 GPa	0 GPa	147 GPa	0 GPa	0 GPa
0 GPa	0 GPa	0 GPa	0 GPa	147 GPa	0 GPa
0 GPa	0 GPa	0 GPa	0 GPa	0 GPa	147 GPa
